# The Impact of Natural Selection on Short Insertion and Deletion Variation in the Great Tit Genome

**DOI:** 10.1093/gbe/evz068

**Published:** 2019-03-29

**Authors:** Henry J Barton, Kai Zeng

**Affiliations:** Department of Animal and Plant Sciences, University of Sheffield, United Kingdom

**Keywords:** insertions, deletions, distribution of fitness effects, linked selection, adaptive mutation

## Abstract

Insertions and deletions (INDELs) remain understudied, despite being the most common form of genetic variation after single nucleotide polymorphisms. This stems partly from the challenge of correctly identifying the ancestral state of an INDEL and thus identifying it as an insertion or a deletion. Erroneously assigned ancestral states can skew the site frequency spectrum, leading to artificial signals of selection. Consequently, the selective pressures acting on INDELs are, at present, poorly resolved. To tackle this issue, we have recently published a maximum likelihood approach to estimate the mutation rate and the distribution of fitness effects for INDELs. Our approach estimates and controls for the rate of ancestral state misidentification, overcoming issues plaguing previous INDEL studies. Here, we apply the method to INDEL polymorphism data from ten high coverage (∼44×) European great tit (*Parus major*) genomes. We demonstrate that coding INDELs are under strong purifying selection with a small proportion making it into the population (∼4%). However, among fixed coding INDELs, 71% of insertions and 86% of deletions are fixed by positive selection. In noncoding regions, we estimate ∼80% of insertions and ∼52% of deletions are effectively neutral, the remainder show signatures of purifying selection. Additionally, we see evidence of linked selection reducing INDEL diversity below background levels, both in proximity to exons and in areas of low recombination.

## Introduction

Insertion and deletion (INDEL) mutations are an important source of genetic variation, often separated into long and short INDELs due to different calling approaches required for longer variants. There is one short INDEL (here ≤50 bp) for every eight single nucleotide polymorphisms (SNPs) in humans (Montgomery et al. 2013), representing a significant proportion of variation. Short INDELs have been implicated in a range of genomic evolutionary processes, such as the evolution of genome size (Petrov 2002; Hu et al. 2011; Nam and Ellegren 2012; Sun et al. 2012). INDELs arguably contribute more to sequence divergence, in terms of the number of base differences, than SNPs (Britten 2002). Additionally it has been suggested that short INDELs may be instrumental in maintaining an optimal intron size (Parsch 2003; Presgraves 2006).

**Fig. 1.**
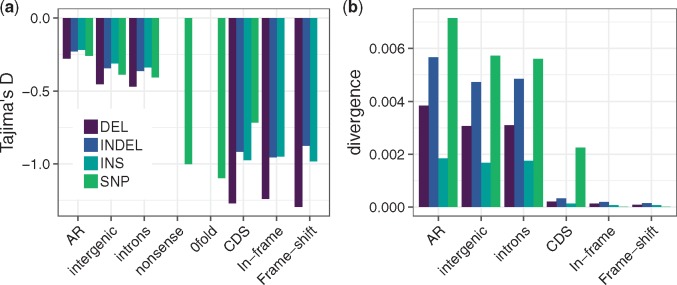
—Tajima’s *D* (a) and divergence (b) estimates for SNPs, INDELs (unpolarized), insertions (INS), and deletions (DEL) in different genomic contexts. Divergence estimates for SNPs are presented as the true divergence divided by 10.

INDEL studies, however, are underrepresented in the literature. In part, this is due to the need to categorize INDELs into insertions and deletions, which requires knowledge of the ancestral state for each variant. This can be obtained using multispecies genome alignments. However, INDELs disproportionately occur in repetitive sequence contexts (Ananda et al. 2013; Montgomery et al. 2013), which are notoriously problematic to align (Earl et al. 2014). Where alignments are successful they are hampered by high rates of ancestral allele misidentification, due to homoplasy. The result is a proportion of deletions are mistakenly identified as insertions (and vice versa), which can confound estimates of selection (Kvikstad and Duret 2014) (see figure 1 in Barton and Zeng [2018]).

Despite the difficulty of analyzing INDEL data, a number of characteristics have been widely reported for INDELs. INDEL mutation is consistently biased toward deletions across a diverse range of organisms (Taylor et al. 2004; Presgraves 2006; Keightley et al. 2009; Hu et al. 2011; Nam and Ellegren 2012; Kvikstad and Duret 2014). Additionally, polymerase slippage has emerged as the predominant force driving short INDEL generation, explaining ∼75% of events in repetitive hotspot regions (Montgomery et al. 2013) and ∼50% of events in nonhotspot regions (Taylor et al. 2004; Montgomery et al. 2013).

In terms of the selective pressures acting on INDELs, deletions consistently segregate at lower frequencies than insertions, both in genes (Sjödin et al. 2010) and genome wide (Chintalapati et al. 2017), which has been interpreted as stronger purifying selection acting on deletions. A mechanistic explanation is that deletions have two breakpoints relative to an insertion’s one, so are more likely to hit an important motif (Petrov 2002; Sjödin et al. 2010). The difference in mean allele frequencies of the two types of variation has also been explained as selection acting on insertions (Ometto et al. 2005). Concordantly, a number of studies have inferred elevated fixation rates for insertions from comparisons of the ratio of deletion to insertion events between polymorphism data and divergence data (Presgraves 2006; Sjödin et al. 2010; Leushkin and Bazykin 2013; Chintalapati et al. 2017). This fixation bias is in line with a number explanations such as selection on insertions to maintain intron lengths (Parsch 2003; Ometto et al. 2005; Presgraves 2006) or insertion-biased gene conversion (Leushkin and Bazykin 2013). However, Kvikstad and Duret (2014) demonstrate the existence of mutation hotspots in repetitive regions, and cryptic hotspots in nonrepetitive regions, which could explain the fixation biases by elevating rates of ancestral state misidentification. They also show that differences in the rate of ancestral misidentification between polymorphism data and divergence data make McDonald–Krietman type tests (McDonald and Kreitman 1991), which in an INDEL context compare polymorphic and fixed numbers of deletions and insertions (e.g., see Chintalapati et al. [2017]), particularly prone to false signatures of fixation bias.

Avian genomes provide a good system for working on INDELs, thanks to their markedly conserved karyotypes and synteny, characterized by having few large macrochromosomes and many smaller microchromosomes (Stapley et al. 2008; Hansson et al. 2010; van Oers et al. 2014; Zhang et al. 2014). Not only does this facilitate genome alignments for ancestral state identification, but also obligate crossing over elevates recombination rates on microchromosomes, driving large intra-genomic variation in recombination (Stapley et al. 2008; Backström et al. 2010; van Oers et al. 2014). This provides power for associating diversity levels with recombination rates. As a result, birds have been the focus of a number of INDEL studies. Nam and Ellegren (2012) propose that high recombination rates drive elevated small deletion rates on microchromosomes and might have caused genome contraction along the lineage leading to birds. Additionally, Rao et al. (2010) show a positive correlation between INDEL density and recombination rate in chicken (*Gallus gallus*) introns. Although this may suggest the impact of linked selection, the use of unpolarized INDEL data means it cannot be distinguished from the impact of a recombination driven mutational bias, such as proposed by Nam and Ellegren (2012). Furthermore, previous work has been constrained by utilizing partial sequencing approaches and neutral markers, negating the formation of a genome-wide picture of INDEL diversity (Brandstrom and Ellegren 2007; Rao et al. 2010; Nam and Ellegren 2012). Thus, despite the advantages of an avian system, the role of natural selection in shaping INDEL diversity in birds is poorly resolved.

Most existing work looking at selection on INDELs has relied upon approaches susceptible to the confounding effects of ancestral state misidentification. There also has been little effort to directly infer unbiased selection coefficients for INDELs, in different genomic contexts. To bridge this gap, we recently published our maximum likelihood model “anavar” for estimating the mutational and selective parameters for INDELs, while simultaneously estimating and controlling for ancestral state misidentification and the confounding effects of demography (Barton and Zeng 2018). Here, we apply this approach to INDEL polymorphism data from 10 European great tit (*Parus major*) genomes from Corcoran et al. (2017). We investigate the selective pressures acting on INDELs across the great tit genome and estimate selection coefficients and the proportion of substitutions fixed by positive selection (*α*) in coding regions. We also seek to address how INDEL diversity changes with distance from coding regions and assess the impact of linked selection on INDEL variation, an area understudied in the literature so far. The great tit genome is particularly well positioned to address these questions with an abundance of current genomic resources available including a well annotated reference genome, high coverage resequencing data, and replicated linkage maps (van Oers et al. 2014; Laine et al. 2016; Corcoran et al. 2017).

## Materials and Methods

### The Great Tit Data Set

The great tit data set consisted of ten European males (1280, 1485, 15, 167, 249-R, 318, 61, 917, 943-R, and TR43666) from a subset of sampling locations in Laine et al. (2016) as described in Corcoran et al. (2017). The mean coverage of the sample is 44×.

### Data Preparation and Variant Calling

Base quality score recalibrated and INDEL realigned BAM files, and an all-sites VCF file containing raw variant calls produced by GATK (version 3.4) (McKenna et al. 2010; DePristo et al. 2011; Van der Auwera et al. 2013) were obtained from Corcoran et al. (2017).

Variant quality score recalibration (VQSR) was then performed for INDELs. This step requires a set of high confidence variants. To generate this data set, we intersected the raw variants called from GATK with variants called with SAMtools (version 1.2) (Li et al. 2009). The resulting variants were filtered using the GATK best practice hard filters (QD < 2.0, ReadPosRankSum<−20.0, FS > 200.0, see https://software.broadinstitute.org/gatk/guide/article? id=3225; last accessed October 1, 2018). Variants with coverage more than twice, or less than half, the mean coverage of 44× were excluded, along with variants falling in repeat regions identified by RepeatMasker (Smit et al. 2013). INDELs with more than two alleles of different length (multiallelic sites) were excluded and INDELs >50 bp. Post-VQSR, we retained variants that fell within the 99% tranche cut-off. The passing variants were then refiltered as above with the exception of the GATK hard filters, which were not reapplied.

For SNPs, variants passing the 99% tranche cut-off in the data set of Corcoran et al. (2017) were obtained and subject to the same post-VQSR hard filters as described above for INDELs.

### Multispecies Alignment and Polarization

We created a multispecies alignment between zebra finch (*Taeniopygia guttata*) (Warren et al. 2010) (version: TaeGut3.2.4, available from: ftp://ftp.ensembl.org/pub/release-84/fasta/taeniopygia_guttata/dna/; last accessed October 1, 2018), flycatcher (*Ficedula albicollis*) (Ellegren et al. 2012) (version: FicAlb1.5, available from: http://www.ncbi.nlm.nih.gov/genome/? term=flycatcher; last accessed October 1, 2018) and great tit (version 1.04) (Laine et al. 2016) with the MULTIZ package (Blanchette et al. 2004) per chromosome, following the pipeline described in Corcoran et al. (2017).

The ancestral states of each variant were then inferred using a parsimony approach where all outgroups were required to match either the reference, or the alternate, allele in the great tit in order to assign it as ancestral.

### Variant Annotation

All variants were annotated as coding, intronic, or intergenic using the great tit annotation (version 1.03) (available from: ftp://ftp.ncbi.nlm.nih.gov/genomes/all/GCF/001/522/545/GCF_001522545.1_Parus_major1.0.3/GCF_001522545.1_Parus_major1.0.3_genomic.gff.gz; last accessed October 1, 2018). Additionally the possible locations of 4-fold degenerate sites, 0-fold degenerate sites and nonsense mutations were identified using the great tit coding sequence (CDS) fasta file (version 1.03) (available from: ftp://ftp.ncbi.nlm.nih.gov/genomes/all/GCF/001/522/545/GCF_001522545.1_Parus_major1.0.3/GCF_001522545.1_Parus_major1.0.3_cds_from_genomic.fna.gz; last accessed October 1, 2018). SNPs at these positions were then identified.

We identified ancestral repeats (ARs, specifically, LINEs) by intersecting the RepeatMasker coordinates for each species with our whole genome alignment and identifying positions annotated as LINEs in all three species. Variants within these regions were identified from the VCF files prior to filtering and were then filtered as described previously, with the exception of the repeat filtering.

We identified callable sites for use in the calculation of summary statistics and our anavar analyses by applying our filters to the original all-sites VCF file and restricting the sites to those that we could polarize.

### Summary Statistics

We calculated nucleotide diversity (*π*) (Tajima 1983) and Tajima’s *D* (Tajima 1989) for INDELs and SNPs both genome-wide and in ARs, introns, intergenic regions, and CDS. In coding regions, we analyzed mutations that preserve the reading frame (in-frame: SNPs, and INDELs a multiple of three in length) and those that shift the reading frame (frameshift: remaining INDELs) separately. For SNPs, we also calculated these statistics for 4-fold degenerate sites, 0-fold degenerate sites, and nonsense mutations. Additionally, we calculated Tajma’s *D* for each INDEL length group separately. Note that although classically *π* refers to the average number of nucleotide differences (Tajima 1983), for INDELs we are measuring the average number of mutation differences without accounting for the number of bases a given INDEL encompasses.

We also calculated Tajima’s *D* and *π* using the site frequency spectrum corrected for orientation errors. We took the model estimates of polarization error for the regions under consideration (see supplementary table S1, Supplementary Material online), and solved the system of linear equations:

(1) $$\varphi_{i}^{\text{ins},\text{obs}}=(1-\epsilon^{\text{ins}})\varphi_{i}^{\text{ins}}+\epsilon^{\text{del}}\varphi_{n-i}^{\text{del}},$$

(2) $$\varphi_{n-i}^{\text{del},\text{obs}}=(1-\epsilon^{\text{del}})\varphi_{n-i}^{\text{del}}+\epsilon^{\text{ins}}\varphi_{i}^{\text{ins}},$$

for 1≤i<n, where $\varphi_{i}^{\text{ins},\text{obs}}$ ($\varphi_{i}^{\text{del},\text{obs}}$) is the observed number of insertions (deletion) of frequency *i*, *ϵ*^ins^ (*ϵ*^del^) the probability that the ancestral state of an insertion (deletion) is incorrectly identified, and $\varphi_{i}^{\text{ins}}$ ($\varphi_{i}^{\text{del}}$) the underlying (unobserved) site frequency spectrum for insertions (deletions). Tajima’s *D* and *π* were then calculated using $\varphi_{i}^{\text{ins}}$ and $\varphi_{i}^{\text{del}}$.

We calculated the distribution of INDEL lengths from our VCF file, both genome-wide and in CDS regions. Within CDS regions, we calculated the proportion of in-frame INDELs per gene. We calculated this proportion both for all genes and for a set of conserved genes identified in Corcoran et al. (2017).

Divergence estimates for INDELs were calculated by counting the number of fixation events unique to the great tit lineage in our whole genome alignment, and dividing by the number of sites that were aligned in all three species for each region analyzed (CDS, AR, intron, and intergenic). For SNPs, we created concatenated FASTA files for each region (CDS, AR, intron, and intergenic), and obtained a pairwise distance matrix using APE (Paradis et al. 2004) in R (R Core Team 2015). The pairwise distance estimates were then used to get an estimate for the branch leading to the great tit.

### Distribution of Fitness Effect Analysis

To estimate the distribution of fitness effects (DFEs) for INDELs, we used the “neutralINDEL_vs_selectedINDEL” model in the anavar package (Barton and Zeng 2018) (available from: http://zeng-lab.group.shef.ac.uk/wordpress/? page_id=28; last accessed October 1, 2018). The package controls for the confounding effects of polarization error and demography (Barton and Zeng 2018). We fitted two types of models for the DFE. The first type fits a discrete number of site classes (*c*) to the data, each class having its own scaled selection coefficient, $\gamma =4N_{e}s$. The per-site scaled mutation rate, $\theta =4N_{e}\mu$, may be equal across sites (the equal mutation rate model), or be different between the neutral sites and the focal sites (the variable mutation rate model). Finally, the model has polarization error parameters, *ϵ*^ins^ and *ϵ*^del^, for both insertions and deletions. The second type of model is similar but assumes continuous gamma distributions for the selection coefficients for INDELs. Different variants of these two types of model were fitted (e.g., with different numbers of site classes and with the mutation rate being either equal or variable) and were compared using Akaike information criterion (AIC).

We used INDELs in ARs (as described previously) as neutral reference and applied the models separately to CDS INDEL data and to noncoding INDEL data. For CDS data, we assumed the equal mutation rate model. This is necessary in order to estimate the proportion of substitutions fixed by positive selection (*α*), as well as estimating the proportion of strongly deleterious variants that do not contribute to polymorphism. We calculated *α* using equation (19) from Barton and Zeng (2018). For noncoding data we employed the variable mutation rate model, which fitted the data better than the equal mutation rate model. We will explore the effects of model choice on our results in the Discussion.

### Exon Proximity Analysis

To investigate the impact of linked selection on INDEL diversity patterns in regions adjacent to CDS, we extracted INDELs and numbers of callable sites in 2-kb adjacent windows moving away from exons up to a maximum distance of 100 kb. The data from all windows at each distance were then binned, creating 50 distance bins. We ran each of the resulting data sets through the anavar package. We fitted the “neutralINDEL_vs_selectedINDEL” model with a continuous *γ* distribution and variable mutation rates, as this was the best-fitting model for noncoding INDELs (supplementary table S4, Supplementary Material online). We used the same neutral reference as in our previous analysis. The relationship between the model’s *θ* estimates and distance from exons was tested with Spearman’s correlations using the “cor.test” function in R (R Core Team 2015). We repeated this analysis using *π* estimates for INDELs instead of the model’s mutation rate estimates.

To look at the relative contributions of different selective site classes to INDEL diversity in each window, we separated our *θ* estimates into *θ* for sites with 0≤γ≤1 and *θ* for γ>1 using the model outputs, we repeated the correlation analysis for these data sets.

To assess to what extent the relationship between distance from exon and diversity was driven by bins close to exons, we generated downsized data sets by progressively removing bins, starting by removing the nearest bin, and then the next nearest, and so on, up until only the furthest two bins were left. We reported the Spearman’s correlation coefficient (*ρ*) and the significance for each downsampled data set.

### Recombination Correlation Analysis

To investigate the relationship between local recombination rate and the action of linked selection, we divided the great tit genome into 2-Mb nonoverlapping windows. We extracted noncoding INDEL calls for each window from our VCF file, excluding windows with <500 polarizable INDELs. As we lacked sufficient data to obtain a regional neutral reference for each window, we were unable to apply our model based approach. Instead, we calculate *π* and Tajima’s *D* for each window. We also estimated noncoding INDEL divergence per window as described previously.

Mean recombination rate was estimated per window. This was achieved by estimating a point recombination rate for every INDEL in the window, along with positions 2-kb up- and down-stream of each variant and taking a mean across all these values. The site specific recombination rates were estimated using the pipeline described in Corcoran et al. (2017). Briefly, we fitted third order polynomials as a function of physical position versus map length for each chromosome using the great tit linkage map data (van Oers et al. 2014). The derivative of each chromosome’s polynomial was then used to estimate recombination rate at a given genomic position.

The relationships of Tajima’s *D* and *π* with local recombination rate were analyzed with Spearman’s correlations using the “cor.test” function in R (R Core Team 2015). The relationship between *π* and recombination rate was also analyzed using partial Spearman’s correlations, with divergence estimates as a confounding variable, to control for the mutagenic effect of recombination, using the “ppcor” package (Kim 2015) in R.

### Data Availability

Detailed documentation of the analysis pipeline along with all scripts used is available at https://github.com/henryjuho/parus_indel (last accessed October 1, 2018). The python scripts make use of the pysam python package (https://github.com/pysam-developers/pysam; last accessed October 1, 2018) and the anavar_utils package (https://henryjuho.github.io/anavar_utils/; last accessed October 1, 2018).

## Results

### Summary of the Data Set

Using the high coverage resequencing data from Corcoran et al. (2017), we called polymorphic INDELs and SNPs according to a GATK based pipeline (Van der Auwera et al. 2013). We polarized variants using a custom multispecies genome alignment and a parsimony based approach. Application of our data calling pipeline to the ten European great tit samples yielded 10,259,689 SNPs and 1,162,517 short INDELs (≤50 bp), of which we could polarize 254,040 insertions and 329,506 deletions. This reduction in variants in the polarized data set is mainly a result of gaps in the whole genome alignment and “hotspots” where the INDEL breakpoints differ between species in the alignment (supplementary fig. S1, Supplementary Material online).

Genome-wide diversity (*π*) for INDELs is around 10-fold lower than that for SNPs. This scale of difference between the two forms of variation was found in all genomic regions analyzed other than in CDS regions where INDEL diversity is close to 80 times lower than SNP diversity. Additionally, we see that within INDELs *π* is biased toward deletions in all regions (table 1).

**Table 1:** Nucleotide Diversity (*π*) for SNPs, INDELs (Unpolarized), Insertions (ins), and Deletions (del) in Different Genomic Contexts

Context	*π*	*π*_indel_	*π*_ins_	*π*_del_
Genome wide	0.00310	0.000356	0.000113 (0.000112)	0.000142 (0.000144)
Ancestral repeats	0.00432	0.000363	0.000117 (0.000119)	0.000175 (0.000177)
Intergenic	0.00333	0.000378	0.000121 (0.000119)	0.000154 (0.000157)
Introns	0.00306	0.000361	0.000116 (0.000115)	0.000143 (0.000145)
CDS	0.00145	$1.87\times 10^{-5}$	$3.61\times 10^{-6}$ $\left(4.36\times 10^{-6}\right)$	$5.25\times 10^{-6}$ $\left(5.09\times 10^{-6}\right)$
In-frame	—	$9.43\times 10^{-6}$	$1.71\times 10^{-6}$ $\left(1.86\times 10^{-6}\right)$	$3.00\times 10^{-6}$ $\left(3.04\times 10^{-6}\right)$
Frameshift	—	$9.28\times 10^{-6}$	$1.90\times 10^{-6}$ $\left(2.17\times 10^{-6}\right)$	$2.24\times 10^{-6}$ $\left(2.27\times 10^{-6}\right)$
4-Fold	0.00369	—	—	—
0-Fold	0.000586	—	—	—
Nonsense	$2.45\times 10^{-5}$	—	—	—

Note.—Estimates in parentheses corrected for polarization error.

When considering INDEL sequence length we observe that the length distribution is enriched in shorter variants, with 80% of INDELs <5-bp long. Additionally, within CDS, we note that the length distribution is enriched in variants that are a multiple of three in length, in other words, mutations that preserve the reading frame (in-frame) (supplementary fig. S2, Supplementary Material online). This enrichment is even more pronounced in conserved genes (supplementary fig. S3, Supplementary Material online). To further investigate the differences between in-frame and frameshifting INDELs, we first note that it is far more likely for an INDEL mutation to have a length that is not a multiple of three than otherwise. This can be seen by the fact that, in putatively neutrally evolving AR regions, *π* values for insertions and deletions with lengths not a multiple of three are $9.8\times 10^{-5}$ and $1.4\times 10^{-4}$, respectively, whereas for those with lengths a multiple of three, the values are $1.9\times 10^{-5}$ and $3.4\times 10^{-5}$. When we consider this in terms of the ratio of AR to CDS diversity (using the CDS *π* values in table 1), for mutations that shift the reading frame we get a ratio of 52 for insertions and 63 for deletions, whereas for in-frame mutations the ratios are both 11. This indicates a much larger reduction in diversity for frameshifting INDELs, and this reduction is more pronounced for deletions, supporting the idea that they are more deleterious.

In general, ARs have the highest diversity level and the least negative Tajima’s *D* for both INDELs and SNPs (table 1 and fig. 1*a*). This supports our decision to use them as a putatively neutral reference in the subsequent analyses. The fact that Tajima’s *D* values are consistently negative in AR regions (fig. 1*a*) is consistent with a recent population expansion for the great tit, as previously reported (Laine et al. 2016; Corcoran et al. 2017). Intronic and intergenic regions have similar diversity patterns across all mutation types, so we grouped them as “noncoding” in subsequent analyses. Tajima’s *D* values for the unpolarized INDELs in CDS regions are similar to those for 0-fold SNPs and SNPs that cause premature stop codons (nonsense mutations). However when polarized, we see that deletions in CDS regions have the most negative Tajima’s *D* of all (fig. 1*a*). In noncoding regions, Tajima’s *D* is negatively correlated with INDEL size for both insertions (Spearman’s $\rho =-0.95,\;P<2.2\times 10^{-16}$) and deletions (Spearman’s ρ=−0.40, *P *=* *0.0038), suggesting that longer variants are probably more deleterious (supplementary fig. S4, Supplementary Material online). In coding regions, we lack power when subsetting INDELs by length (supplementary fig. S4, Supplementary Material online).

The patterns reported above are mirrored by the divergence estimates. The highest divergence is seen in ARs. Intergenic and intronic regions have similar divergence levels, and both have lower divergence than ARs. In CDS regions, divergence is lowest, 14 times lower than the genome-wide average for INDELs. SNP divergence is around 10-fold higher than INDEL divergence in noncoding regions, in line with *π* estimates. In CDS regions, SNP divergence is 70-fold higher than INDEL divergence (fig. 1*b*). These results are robust to polarization error (table 1 and supplementary fig. S5, Supplementary Material online).

### The Distribution of Fitness Effects

To describe the DFEs for INDELs, we fitted four distinct DFEs to coding and noncoding data separately. For coding data, the model assumes equal mutation rates between neutral and focal sites, a requirement to calculate the proportion of substitutions fixed by positive selection (*α*). For noncoding data where *α* was not calculated, this assumption was relaxed and mutation rates were free to vary (see Materials and Methods). The best-fit model for each case is reported in table 2.

**Table 2:** Maximum Likelihood Parameter Estimates for the Best-Fitting Models for INDELs in CDS Regions and Noncoding regions

Model and DFE	Variant Type	*C*	*θ*	*γ*	Scale	Shape	*ϵ*	*α* (%)
CDS: equal mutation rate	Insertions	1	$4.92\times 10^{-6}$	–1.14	—	—	0.0799	
Discrete *C* = 2	Insertions	2	0.000134	–801	—	—	0.000307	71
Ancestral repeat reference	Deletions	1	$8.32\times 10^{-6}$	–2.70	—	—	0.0368	
	Deletions	2	0.000206	–649	—	—	$3.12\times 10^{-7}$	86
CDS: equal mutation rate	Insertions	1	$4.79\times 10^{-6}$	–0.264	—	—	0.0729	
Discrete *C* = 2	Insertions	2	0.000156	–897	—	—	0.000526	63
Noncoding reference	Deletions	1	$7.79\times 10^{-6}$	–1.70	—	—	0.0366	
	Deletions	2	0.000205	–629	—	—	0.00587	79
Noncoding: free mutation rate	Insertions	—	0.000170	–53.6	1,553	0.0345	0.0110	—
Continuous	Deletions	—	0.000293	–75.5	715	0.106	0.0166	—

Note.—*C* defines the number of site class, *θ* the population scaled mutation rate, *γ* the population scaled selection coefficient, *ϵ* the polarization error, and *α* the proportion of INDEL substitutions driven by positive selection. Where *γ* values are presented for the continuous model these are mean *γ* estimates and the product of the scale and shape parameters.

**Fig. 2.**
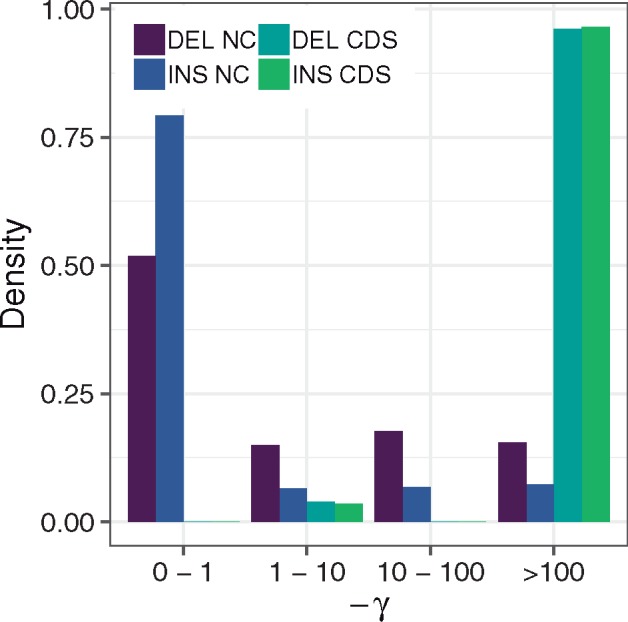
—DFEs for noncoding insertions (INS NC), noncoding deletions (DEL NC), coding insertions (INS CDS), and coding deletions (DEL CDS), shown as the proportion of mutations falling into different selection coefficient (*γ*) bins.

The best-fit INDEL DFE (according to AIC, see supplementary table S2, Supplementary Material online) in coding regions is bimodal, characterized by a class of strongly deleterious INDELs making up 96% of sites and a class of weakly deleterious INDELs for the remaining 4% of sites (fig. 2). For those variants with weakly negative *γ* estimates (i.e., those segregating in our sample), deletions are more deleterious, however for the strongly deleterious class of INDELs, insertions have the more negative selection coefficient. We subsequently estimate the proportion of INDEL substitutions fixed by positive selection (*α*) at 71% for insertions and 86% for deletions (table 2). When we run this analysis using a noncoding neutral reference we recapture a very similar bimodal DFE, but with slightly lower *α* values, 63% for insertions and 79% for deletions (table 2 and supplementary table S3, Supplementary Material online).

The noncoding INDEL data are best fit by a continuous gamma DFEs (supplementary table S4, Supplementary Material online). We see small shape parameter estimates of 0.0345 for insertions and 0.106 for deletions (table 2), describing a DFE enriched in effectively neutral variants. When binning this gamma distribution into four −γ categories (0–1, 1–10, 10–100, and >100) we see that ∼80% of insertions and ∼52% of deletions in noncoding regions have *γ* estimates between 0 and –1 and can be considered as effectively neutral. The remaining proportions of variants are evenly distributed between the other three selective categories (fig. 2). For noncoding and coding data, there is a marked deletion bias with the deletion to insertion ratio estimated at 1.5 in coding regions and 1.7 in noncoding regions.

### The Impact of Linked Selection

To test for evidence of linked selection acting on INDELs, we obtained estimates of the scaled INDEL mutation rates (*θ*_ins_ and *θ*_del_, respectively) in 2-kb nonoverlapping bins with increasing distance from exons, up to 100 kb away.

**Fig. 3.**
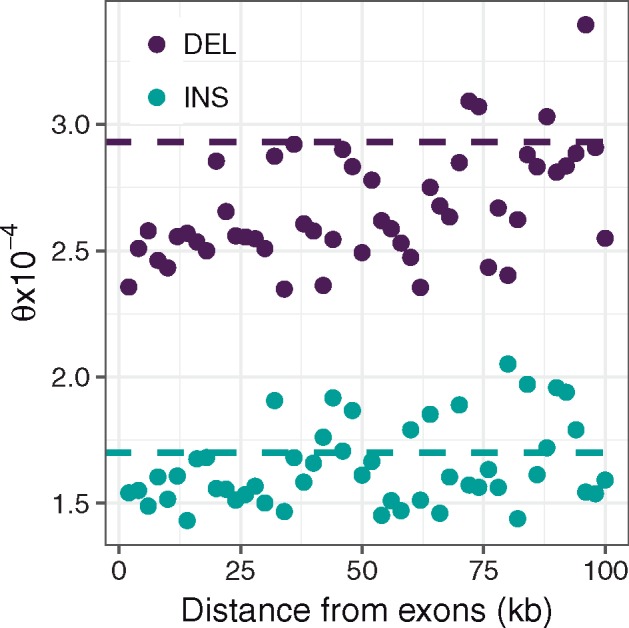
—Relationship between mutation rate estimates (*θ*) for insertions (turquoise) and deletions (purple) and distance from exons in 2-kb windows. Dashed lines represent the genome-wide average mutation rate for noncoding variants, as shown in table 2.

We find significant positive correlations between our model estimates of both *θ*_del_ (Spearman’s ρ=0.47, *P *=* *0.00058) and *θ*_ins_ (Spearman’s *ρ *= 0.28, *P* = 0.046) with distance from exons (fig. 3). This relationship is corroborated when using *π* estimates for deletions and insertions (deletions: Spearman’s $\rho =0.79,\;P=2.2\times 10^{-16}$, insertions: Spearman’s $\rho =0.84,\;p=2.2\times 10^{-16}$, see supplementary fig. S6, Supplementary Material online). We separated variants into two *γ* ranges, 0 to –1 and <−1 and reanalyzed this relationship. For the putatively neutral sites, we recapture this significant correlation between *θ* and distance from exons (*θ*_del_: Spearman’s $\rho =0.54,\;P=7.9\times 10^{-5}$, *θ*_ins_: Spearman’s $\rho =0.57,\;P=2.3\times 10^{-5}$). However, for the more deleterious category, we see no relationship (*θ*_del_: Spearman’s ρ=−0.027, *P *=* *0.85, *θ*_ins_: Spearman’s ρ=−0.15, *P *=* *0.30) (supplementary fig. S7, Supplementary Material online). Additionally, to assess how these correlations held up when using data further from exons we performed correlations on downsampled data sets by cumulatively removing each bin nearest to exons in turn, progressively reducing our number of bins from 50 to 2. We see that for *π* we recover significant positive correlations (for both deletions and insertions) for data sets starting up to ∼35 kb from exons. For *θ*, we recover this relationship for deletions up to ∼40 kb from exons, however for insertions, we lack statistical power from the model estimates, probably due to there being relatively fewer insertion polymorphisms (supplementary fig. S8, Supplementary Material online).

### Recombination Rate and INDEL Diversity

To obtain additional evidence for linked selection, we separated our noncoding INDEL data into 322 2-Mb genomic windows, each with a mean recombination rate estimate. As a lack of a regional neutral reference per window precluded the use of our model, we instead obtained estimates of *π* and Tajima’s *D* for each window.

**Fig. 4.**
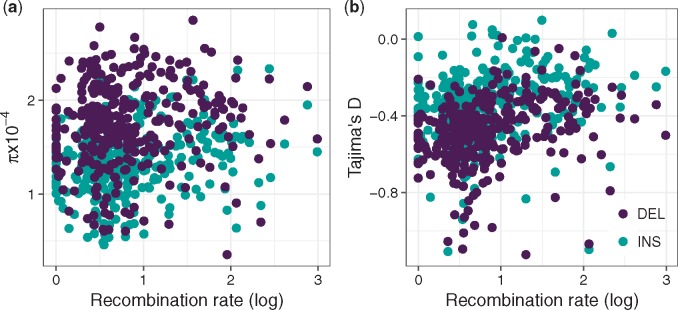
—The relationship between local recombination rate (log transformed) and *π* (*a*) and Tajima’s *D* (*b*) for both insertions (turquoise) and deletions (purple).

We report positive relationships between *π*_ins_ and recombination rate (Spearman’s ρ=0.18, *P *=* *0.0010), and *π*_del_ and recombination rate (Spearman’s ρ=0.12, *P *=* *0.027) (fig. 4*a*). However, when introducing INDEL divergence as a covariate in a partial correlation analysis (to control for the possible mutagenic effects of recombination), we only maintain the relationship between *π*_ins_ and recombination rate (partial Spearman’s ρ=0.15, *P *=* *0.0076) and not *π*_del_ (partial Spearman’s ρ=0.077, *P *=* *0.17). Additionally, we see a significant enrichment of low frequency variants in low recombining regions, as measured by Tajima’s *D*, for both insertions (Spearman’s $\rho =0.30,\;P=3.7\times 10^{-8}$) and deletions (Spearman’s $\rho =0.33,\;P=1.5\times 10^{-9}$) (fig. 4*b*).

## Discussion

INDELs often remain unanalyzed in sequencing studies, despite constituting a large proportion of genetic variation (Brandstrom and Ellegren 2007; Montgomery et al. 2013). This is largely a result of the difficulty of working with INDELs compared with SNPs (see Introduction). Yet, when INDELs do get analyzed, studies are hampered by the issue of ancestral state misidentification confounding signatures of selection (Kvikstad and Duret 2014), leaving the selective landscape for INDELs poorly defined. Here, we seek to overcome this hurdle using our recently published model (Barton and Zeng 2018), to estimate the DFE for INDELs in an avian genome. We use high coverage resequencing data from ten European great tits from Corcoran et al. (2017), to quantify the levels of purifying and positive selection for INDELs in coding regions and report evidence of linked selection acting on noncoding INDELs.

### Coding Sequence INDELs

The majority of INDELs in our data set are <5 bp in length. The most common length is 1-bp genome wide, but 3 bp within coding regions (supplementary fig. S2, Supplementary Material online). This enrichment of in-frame INDELs is even more pronounced in conserved genes (supplementary fig. S3, Supplementary Material online). Consistently, we report that frameshifting INDELs have a more severe reduction in diversity and more negative Tajima’s *D* than in-frame INDELs. In noncoding regions, we see strong negative correlations between INDEL length and Tajima’s *D*. Taken together, these results provide confidence in the genome annotation, show the importance of INDEL length in coding regions with frameshifting INDELs more deleterious, and provide evidence that longer noncoding INDELs are more deleterious. These results are consistent with previous studies (Sjödin et al. 2010; Montgomery et al. 2013; Barton and Zeng 2018).

From the application of our model, we see that the majority (96%) of deletions and insertions occurring in CDS regions are strongly deleterious (γ<−100) (table 2 and fig. 2). This proportion corresponds to our previous estimates for INDELs in *Drosophila melanogaster* of between 92% and 97% (Barton and Zeng 2018). Additionally, our values are similar to those reported for SNPs in a number of organisms, including 0-fold degenerate (0-fold) SNPs in the great tit (∼80% with γ<−10) and zebra finch (*Taeniopygia guttata*) (∼85% with γ<−10) (Corcoran et al. 2017), and nonsynonymous SNPs in *D. melanogaster* (78% with γ<−100) and *Mus musculus castaneus* (69% with γ<−100) (Kousathanas and Keightley 2013). We estimate the proportion of INDEL substitutions fixed by positive selection, *α*, at 86% for deletions and 71% for insertions (or 79% and 63%, respectively, when using noncoding INDELs as neutral reference) (table 2). This is comparable to our previous estimates of *α* for deletions (81%) and insertions (60%) in *D. melanogaster* (Barton and Zeng 2018), and *α* estimates for SNPs in *D. melanogaster* of between 74% and 95% (Schneider et al. 2011). However, our estimates are higher than the *α* estimate for 0-fold SNPs of 48% obtained by Corcoran et al. (2017) using the same great tit data set. This may reflect stronger purifying selection acting on INDELs than SNPs (in line with our Tajima’s *D* and divergence estimates), which provides a stronger opposing force to genetic drift and hence reduces the number of INDEL fixations by drift relative to SNPs. Both our *γ* estimates for weakly selected sites and *α* estimates point to deletions being more deleterious than insertions, in line with theoretical expectations that deletions impact more sequence than insertions, and are thus more likely to hit an important motif (Petrov 2002; Sjödin et al. 2010), as reported in other studies (Sjödin et al. 2010; Montgomery et al. 2013; Chintalapati et al. 2017).

A number of potential caveats are worth noting however. First, the great tit has likely experienced a recent population expansion (Laine et al. 2016; Corcoran et al. 2017), consistent with our negative Tajima’s *D* values across the genome. Population expansion can lead to an excess of weakly deleterious fixations relative to the amount seen in polymorphism data, which can artificially inflate estimates of the proportion of mutations fixed by positive selection (Eyre-Walker 2002; Eyre-Walker and Keightley 2009). Here, we have used the method of Eyre-Walker et al. (2006) to control for demography. Existing evidence suggests that this approach is effective in alleviating biases on the estimation of selection intensity on weakly selected variants caused by demography (see fig. 4*a* in Jackson et al. [2017]). Because the best-fitting model suggests that the DFE for both insertions and deletions in coding regions is bimodal, with segregating variants subject to weak purifying selection (table 2), our *α* estimates should be robust.

Second, the formula for estimating *α* (e.g., eq. 19 in Barton and Zeng 2018) assumes that the mutation rate is the same between the neutral reference and the focal sites. For this reason, we employed the equal mutation rate model in our analysis of the coding INDELs. However, we note that the model that assumes a gamma DFE and allows the neutral sites and the coding sites to have different mutation rates fits the data better than the equal mutation rate model presented in table 2 [ΔAIC = AIC(best-fitting equal mutation rate model) − AIC(best-fitting variable mutation rate model) = 4.50]. As demonstrated in Barton and Zeng (2018), this difficulty can be readily alleviated if we know both the point mutation rate and the INDEL mutation rate, which is currently unavailable for the great tit, but can be obtained by direct sequencing of parents and offspring. It should also be noted that both models lead to similar conclusions regarding the DFE. To see this, we calculate p(|X|≤x) for *x *=* *1.5, 5, and 10, where |X| follows a gamma distribution. Using the MLEs (supplementary table S5, Supplementary Material online), for insertions, the proportions are 0.12, 0.18, and 0.23, whereas for deletions, they are 0.052, 0.094, and 0.132. These results are congruent with those shown in table 2 as they indicate that, in coding regions, deletions tend to be under stronger purifying selection, and that only a small fraction INDEL mutations are sufficiently weakly selected that they contribution to observed polymorphism.

Third, as repetitive regions of the genome are notoriously difficult to call variants in and align (Earl et al. 2014), it is possible that our elevated diversity and divergence estimates in ARs could be the result of an increased number of false positive calls in these regions. To assess the impact of our choice of neutral reference on the DFE, we reran our coding analysis using noncoding INDELs as neutral reference. We find that the use of either neutral reference results in a very similar bimodal DFE, with a majority of INDELs being strongly deleterious, and a minority weakly deleterious (table 2). With noncoding INDELs as neutral reference, we observe a slight reduction in the estimated selection pressure on the weakly deleterious site class. This is probably due to the presence of weakly selected variants in the noncoding data set, as we have previously shown (see supplementary table S2 in Barton and Zeng 2018). As the fixation rate is higher when the estimated selection coefficient is smaller, our *α* estimates are also lower in this case, but are still well above zero. Overall, it seems that our use of ARs as neutral reference does not unduly impact our results.

### Noncoding INDELs and Linked Selection

The DFE for noncoding INDELs is best described by a gamma distribution. The shape parameter estimates we obtain for both insertions and deletions are small (0.0345 and 0.106, respectively, table 2), corresponding to 76% of insertions and 52% of deletions having *γ* values between 0 and –1, and thus effectively neutral (fig. 2). The proportion of neutral insertions in noncoding regions (76%) is comparable to the proportion of intronic SNPs with *γ* estimates between 0 and –1 (70%) in *D. melanogaster* (Eyre-Walker and Keightley 2009). However, the proportion of deletions falling into this selective range is markedly lower at 52%, more in line with SNPs in untranslated regions in birds, where in the great tit ∼50%, and in the zebra finch ∼40% of variants fall within the 0 to –1 *γ* range (Corcoran et al. 2017). This mirrors and reinforces the trend seen in coding regions supporting the more deleterious nature of deletions. It also suggests that overall a substantial proportion of INDELs (24% of insertions and 48% of deletions) in noncoding regions are experiencing purifying selection.

To understand how noncoding INDEL diversity changes around coding regions, we investigated how *θ* varies with distance from exons. Our analysis shows that noncoding *θ* estimates adjacent to exons are lower than the genome-wide noncoding estimates. As distance from exons increases, both *θ*_ins_ and *θ*_del_ increase significantly returning to the genome-wide level by 100 kb from exons (fig. 3). As the scaled mutation rate ($\theta =4N_{e}\mu$) is the product of the per-site mutation rate (*μ*) and the effective population size (*N*_e_) changes in *θ* can be the result of changes in either parameter. However, as we do not expect there to be a systematic variation in *μ* between our distance bins, changes in *θ* should be driven by corresponding changes in *N*_e_. This relationship between distance and *θ* could be explained through increasing proximity to functional sequence, and therefore increased linkage to sites either under purifying or positive selection, resulting in reduced *N*_e_ close to exons (see Cutter and Payseur [2013] for review). Alternatively, it could be driven by a higher density of regulatory elements under selective constraint in noncoding sequence near exons, making INDELs closer to exons more deleterious, and thus reducing diversity in these regions. However, two lines of evidence presented here support the former explanation. First, we can recapture the relationship between INDEL diversity and distance from exons when reanalyzing our data set after removing data up to as much as the nearest 30 kb to exons for *π*_ins_, *π*_del_, and *θ*_del_ (although for *θ*_ins_, we lack statistical power). This demonstrates that the correlation is not solely driven by regions directly neighboring exons, as might be expected if driven by purifying selection on regulatory elements, but extends over larger distances, more indicative of linked selection (supplementary fig. S8, Supplementary Material online). Second, when we analyze nearly neutral variants (−1≤γ≤0) and deleterious variants (γ<−1) separately we see that the relationship between distance from exons and *θ* is driven by a significant increase in nearly neutral variants as distance from exons increases. We see no increase in deleterious variants close to exons as would be expected if regulatory elements were disrupted (supplementary fig. S7, Supplementary Material online). Additionally, this suggests that although a proportion of INDELs in noncoding regions seem to be experiencing negative selection, in agreement with our reported genome-wide noncoding DFE, these variants are not driving the reduction of diversity in proximity to exons.

The possibility of linked selection reducing diversity is further supported by the significant positive correlations we see between local recombination rate and *π*_ins_, *π*_del_, and Tajima’s *D* (fig. 4). Linked selection can be expected to generate such a pattern, with linkage decreasing as recombination rates increase, which should drive higher *π* in high recombining regions (Corcoran et al. 2017) and a greater enrichment of low frequency variants in low recombining regions. However, the mutagenic effect of recombination can also be expected to generate relationship between *π* and recombination (Arbeithuber et al. 2015). To disentangle these two forces, we conducted partial correlation analyses using INDEL divergence as a covariate. The partial correlation coefficient between *π*_ins_ and recombination is 0.15, which is significant and close to the value of 0.18 obtained without using divergence as a covariate. In contrast, the partial correlation coefficient between *π*_del_ and recombination rate is 0.077, which is nonsignificant and more different from the value of 0.12 obtained without partial correlation. This suggests that the mutagenic effect of recombination has probably played a role in driving increased INDEL mutation rates in high recombining regions, and that this effect is likely stronger for deletions than insertions. This is in line with results previously reported in zebra finch (Nam and Ellegren 2012). Yet, the greater enrichment in low frequency variants in low recombining regions is not an expected outcome of reduced mutation rates. Thus, it seems likely that the true picture is a combination of both linked selection and mutation variation shaping patterns of INDEL variability in regions of varying recombination.

## Conclusion

In summary, we see that genome-wide INDELs appear to be having detrimental effects, with most coding INDELs strongly deleterious, and a sizeable minority of noncoding INDELs showing signatures of purifying selection. We also show that noncoding INDEL diversity is constrained through linkage to selected sites near exons and in low recombining regions, though some of this can be attributed to the mutagenic effect of recombination. However, we cannot separate how much of this trend is driven by positive selection and how much is due to purifying selection, which would be an interesting avenue for future INDEL studies.

## Supplementary Material

Supplementary data are available at Genome Biology and Evolution online.

## Supplementary Material

Supplementary_Material_evz068
