# Supplementary material for: The Impact of Natural Selection on Short Insertion and Deletion Variation in the Great Tit Genome
Source: Genome Biol Evol. 2019 Mar 29;11(6):1514–24. doi: 10.1093/gbe/evz068 (PMC6543879; doi:10.1093/gbe/evz068)
Supplement: Supplementary_Material_evz068 [file supplementary_material_evz068.pdf]

Table S1 Estimates of polarisation error used to correct the site frequency spectrum prior to calculating the summary statistics. CDS estimates are from the best fit model in table S2, from the weakly deleterious site class, the ancestral repeat (AR) estimates are also from this model. Non-coding estimates are from the best fit model in table S3.

Region	ε_{ins}	ε_{del}
CDS	0.0799	0.0368
Non-coding	0.0110	0.0166
AR	0.0302	0.0261

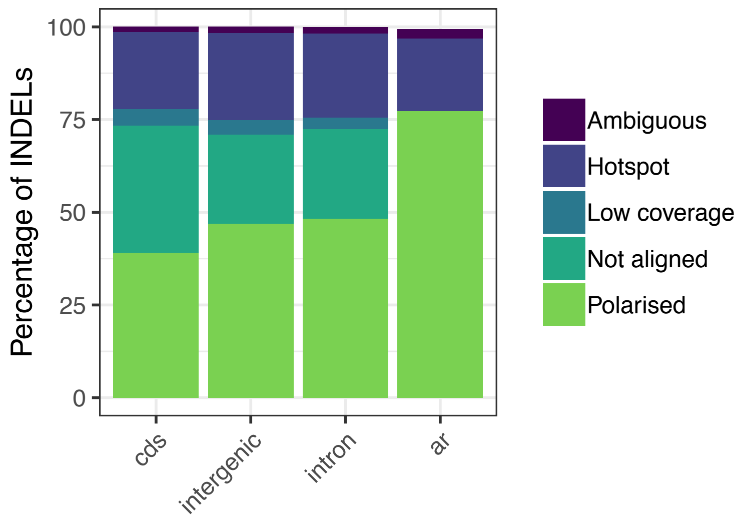

Figure S1 Proportion of INDELs that were polarised in different genomic regions. 'Ambiguous' refers to sites where both great tit alleles are represented in the outgroups, for example due to ancestral polymorphism. Note that due to the way ancestral repeats (ar) were identified from the genome alignment, it is not possible for INDELs to fail polarisation due to being 'not aligned' or have 'low coverage'.

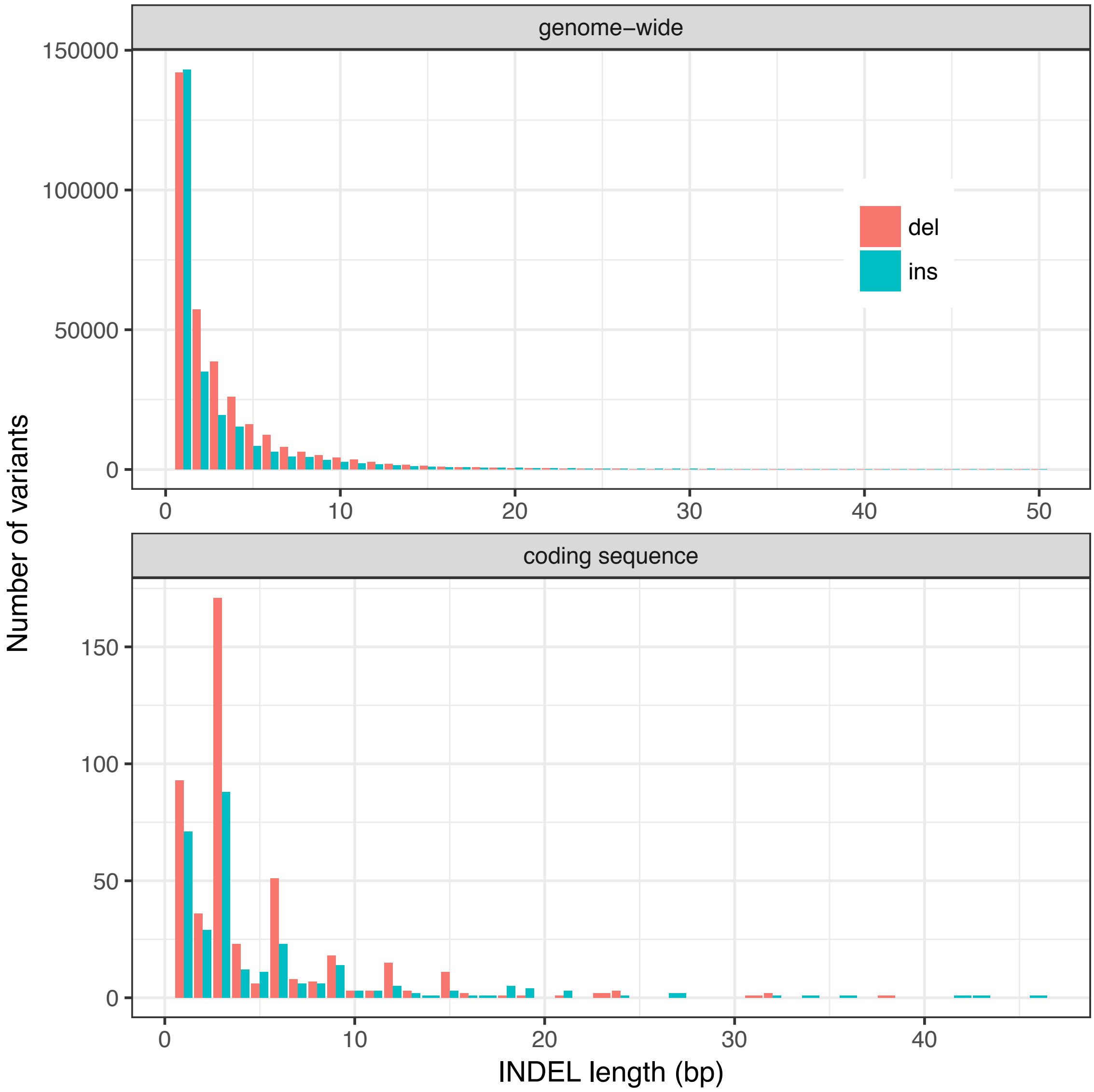

Figure S2 Length distribution of short INDELs (50bp or less) both genome-wide and in coding sequence within the great tit genome.

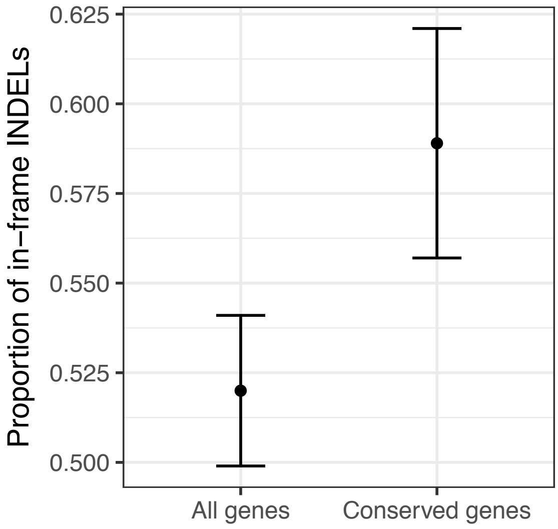

Figure S3 Proportion of INDELs that preserve the reading frame (INDELs a multiple of 3 in length) in all genes compared to conserved genes.

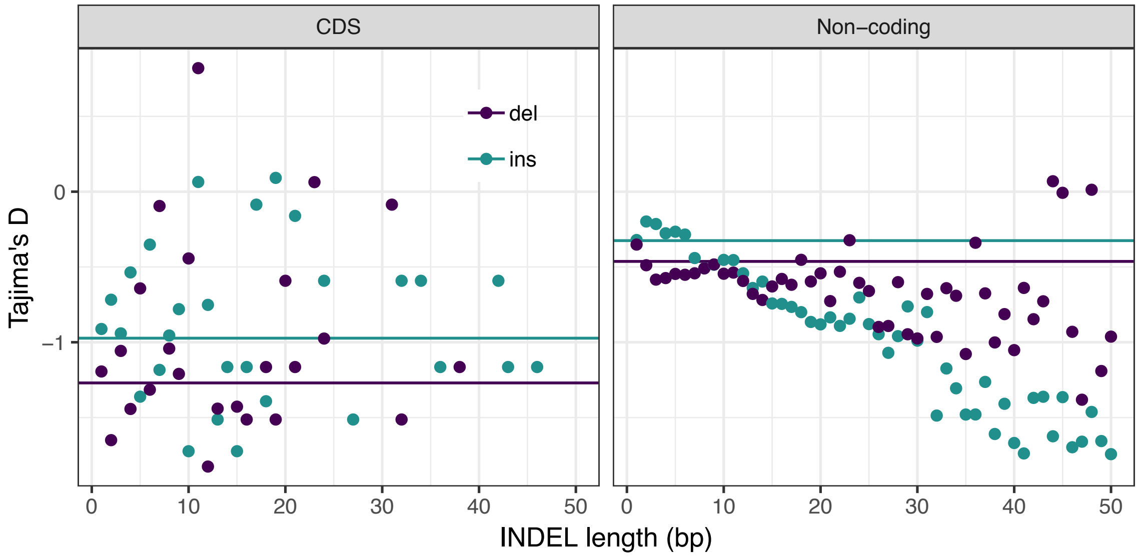

Figure S4 Tajima's *D* for insertions and deletions of different lengths in both coding (CDS) and non-coding regions. Horizontal lines represent the estimates when not separated by length.

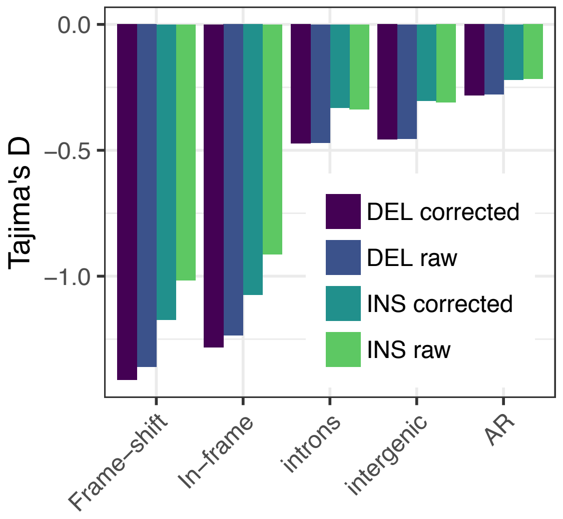

Figure S5 Tajima's *D* estimates for insertions and deletions in different genomic contexts, before (raw) and after correcting for polarisation error (corrected).

Table S2 Maximum likelihood parameter estimates for models assuming equal mutation rates between neutral and focal sites, fitted to coding sequence INDELs with INDELs in ancestral repeats as neutral reference. Where *ΔAIC = AIC(best model) – AIC(lower ranked model).*

Model	Variants	*C*	θ	γ	scale	shape	ε	α	*ΔAIC*
Discrete *C=2*	insertions	1	4.92×10^{-6}	-1.14	-	-	0.0799		
	insertions	2	0.000134	-801	-	-	0.000307	71%	
	deletions	1	8.32×10^{-6}	-2.70	-	-	0.0368		
	deletions	2	0.000206	-649	-	-	3.12×10^{-7}	85%	0
Discrete *C=3*	insertions	1	7.32×10^{-7}	10000	-	-	0.00144		
	insertions	2	0.000141	-748	-	-	0.0870		
	insertions	3	4.04×10^{-6}	-2.56	-	-	4.81×10^{-5}	91%	
	deletions	1	1.17×10^{-6}	83.3	-	-	0.00137		
	deletions	2	9.70×10^{-6}	-5.94	-	-	0.0297		
	deletions	3	0.000208	-859	-	-	0.000368	99%	-5.17
Continuous	insertions	1	0.000130	-1498	2284	0.656	0.0685	100%	
	deletions	1	0.000204	-1551	2326	0.667	0.0442	100%	-106
Discrete *C=1*	insertions	1	0.000103	-37.4	-	-	0.149	100%	
	deletions	1	0.000171	-70.4	-	-	0.0592	100%	-2741

Table S3 Maximum likelihood parameter estimates for models assuming equal mutation rates between neutral and focal sites, fitted to coding sequence INDELs with INDELs in non-coding regions as neutral reference. Where *ΔAIC = AIC(best model) – AIC(lower ranked model).*

Model	Variants	*C*	θ	γ	scale	shape	ε	α	*ΔAIC*
Discrete *C=2*	insertions	1	4.79×10^{-6}	-0.264	-	-	0.0729		
	insertions	2	0.000156	-897	-	-	0.000526	63%	
	deletions	1	7.79×10^{-6}	-1.70	-	-	0.0366		
	deletions	2	0.000205	-629	-	-	0.00587	79%	0
Discrete *C=3*	insertions	1	6.59×10^{-6}	-2.23	-	-	0.0202		
	insertions	2	7.91×10^{-5}	-1011	-	-	0.0491		
	insertions	3	7.76×10^{-5}	-2738	-	-	0.453	85%	
	deletions	1	4.90×10^{-6}	0.597	-	-	0.0839		
	deletions	2	1.52×10^{-5}	-21.1	-	-	0.000163		
	deletions	3	0.000195	-2483	-	-	1.43×10^{-5}	100%	-13.8
Continuous	insertions	1	0.000154	-1614	2413	0.669	0.0667	100%	
	deletions	1	0.000209	-1553	2401	0.647	0.0470	100%	-147
Discrete *C=1*	insertions	1	0.000153	-54.9	-	-	0.141	100%	
	deletions	1	0.000208	-75.7	-	-	0.0648	100%	-3210

Table S4 Maximum likelihood parameter estimates for models with mutation rates free to vary between neutral and focal sites, fitted to non-coding INDELs. Where *ΔAIC = AIC(best model) – AIC(lower ranked model).*

Model	Variants	*C*	θ	γ	scale	shape	*ε*	*ΔAIC*
Continuous	insertions	1	0.000170	-53.6	1553	0.0345	0.0110	
	deletions	1	0.000293	-75.5	715	0.106	0.0166	0
Discrete *C=2*	insertions	1	3.23×10^{-5}	4.64	-	-	0.0358	
	insertions	2	0.000122	-1.44	-	-	0.00293	
	deletions	1	0.000113	1.15	-	-	0.00306	
	deletions	2	0.000117	-5.33	-	-	0.0231	-3.43
Discrete *C=3*	insertions	1	1.20×10^{-5}	99852	-	-	0.463	
	insertions	2	0.000141	-0.514	-	-	0.0241	
	insertions	3	0.000282	-879	-	-	8.59×10^{-6}	
	deletions	1	3.26×10^{-5}	4.67	-	-	0.127	
	deletions	2	0.000169	-127	-	-	0.0327	
	deletions	3	0.000172	-1.65	-	-	0.0294	-10.9
Discrete *C=1*	insertions	1	0.000161	-0.204	-	-	0.0584	
	deletions	1	0.000223	-0.831	-	-	0.0451	-131

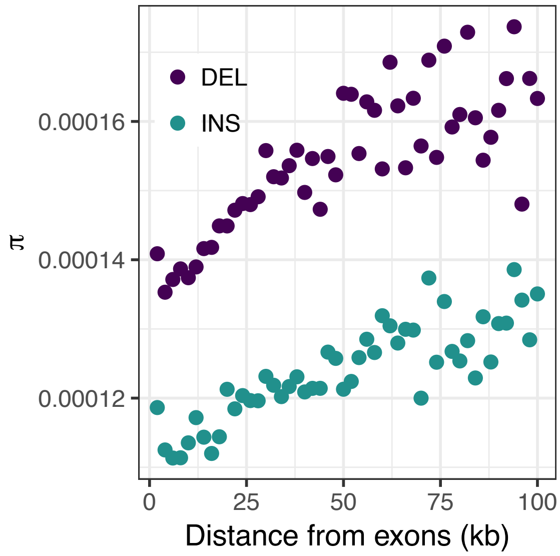

Figure S6 The relationship between INDEL diversity and distance from exons. Each point represents a 2kb bin.

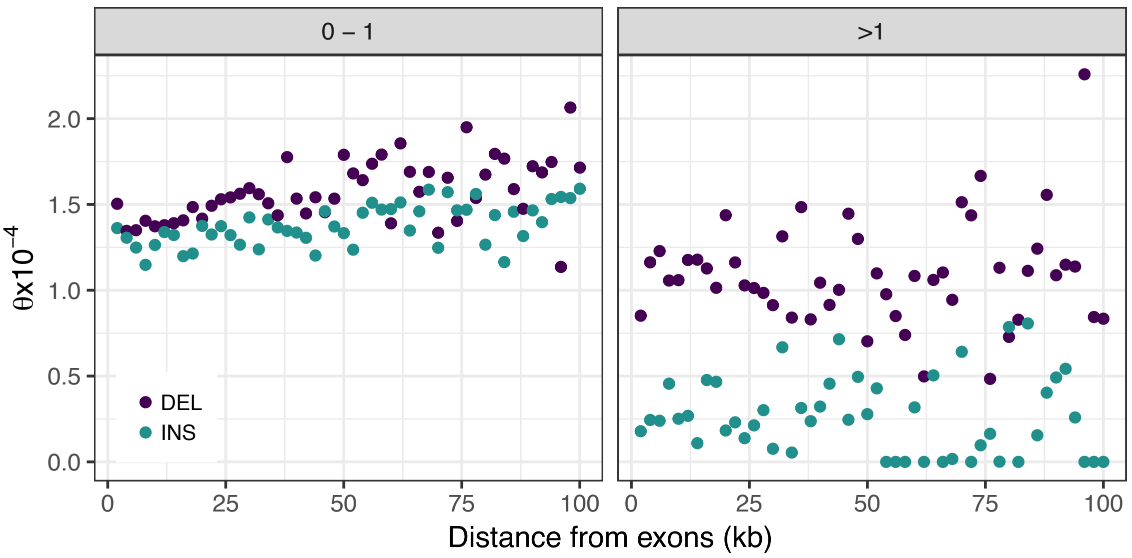

Figure S7 The relationship between θ and distance from exons for putatively neutral INDELs (left panel, -γ between 0 and 1) and negatively selected INDELs (right panel, -γ > 1). Insertions (INS) in turquoise and deletions (DEL) in purple. Each point represents a 2kb bin.

.

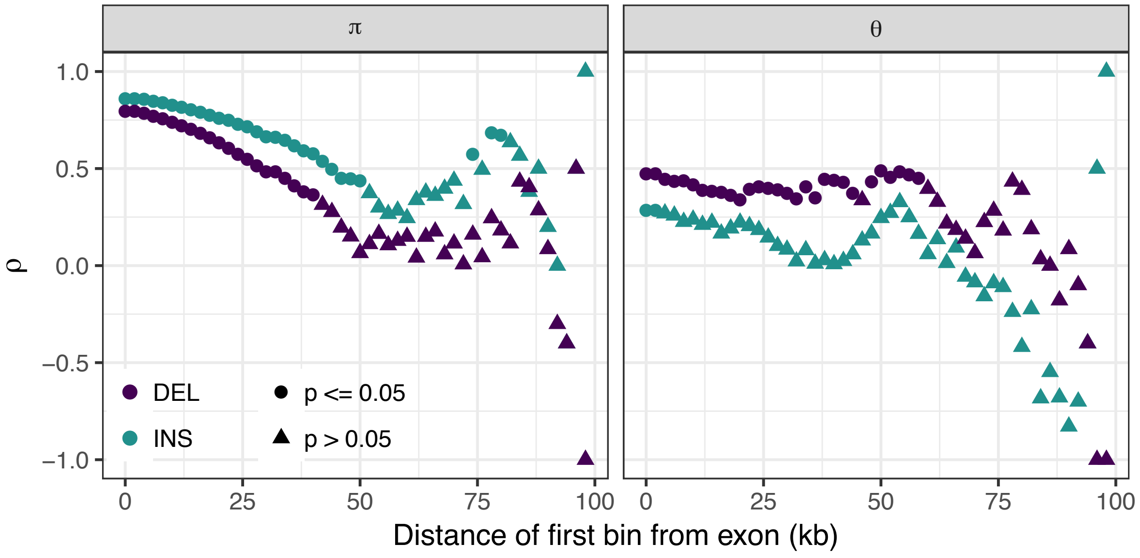

Figure S8 The strength and significance of Spearman's correlations between distance from exons and nucleotide diversity (left) and model based estimates of the scaled mutation rate (right), with increasingly cumulatively down sampled datasets, reduced by iteratively removing the bin nearest to exons for each correlation.

Table S5 Maximum likelihood parameter estimates for the best-fit model with mutation rates free to vary between neutral and focal sites, fitted to coding INDELs**.**

Model	Variants	*C*	θ	γ	scale	shape	ε
Continuous	insertions	1	$2.22 x 10^{-5}$	-336	986	0.341	0.0339
	deletions	1	$5.38 x 10^{-5}$	-374	758	0.494	0.0169
